# Supplementary material for: Multifaceted conserved functions of Notch during post-embryonic neurogenesis in the annelid Platynereis
Source: EMBO Rep. 2026 Apr 1;27(9):2345–68. doi: 10.1038/s44319-026-00731-6 (PMC13172424; doi:10.1038/s44319-026-00731-6)
Supplement: Supplementary file 5 — Appendix [file 44319_2026_731_MOESM5_ESM.pdf]

Appendix

Multifaceted conserved functions of Notch during post-embryonic neurogenesis in the annelid *Platynereis*

Loïc Bideau, Loeiza Baduel, Gabriel Krasovec, Caroline Dalle, Ombeline Lamer, Mélusine Nicolas, Alexandre Couëtoux, Corinne Blugeon, Louis Paré, Michel Vervoort, Pierre Kerner, Eve Gazave

Table of Content

Appendix Figure S1……………………………………………….. 2

Appendix Figure S2……………………………………………….. 3

Appendix Figure S3……………………………………………….. 5

Appendix Figure S4……………………………………………….. 6

Appendix Figure S5……………………………………………….. 7

Appendix Figure S6……………………………………………….. 8

Appendix Figure S7……………………………………………….. 8

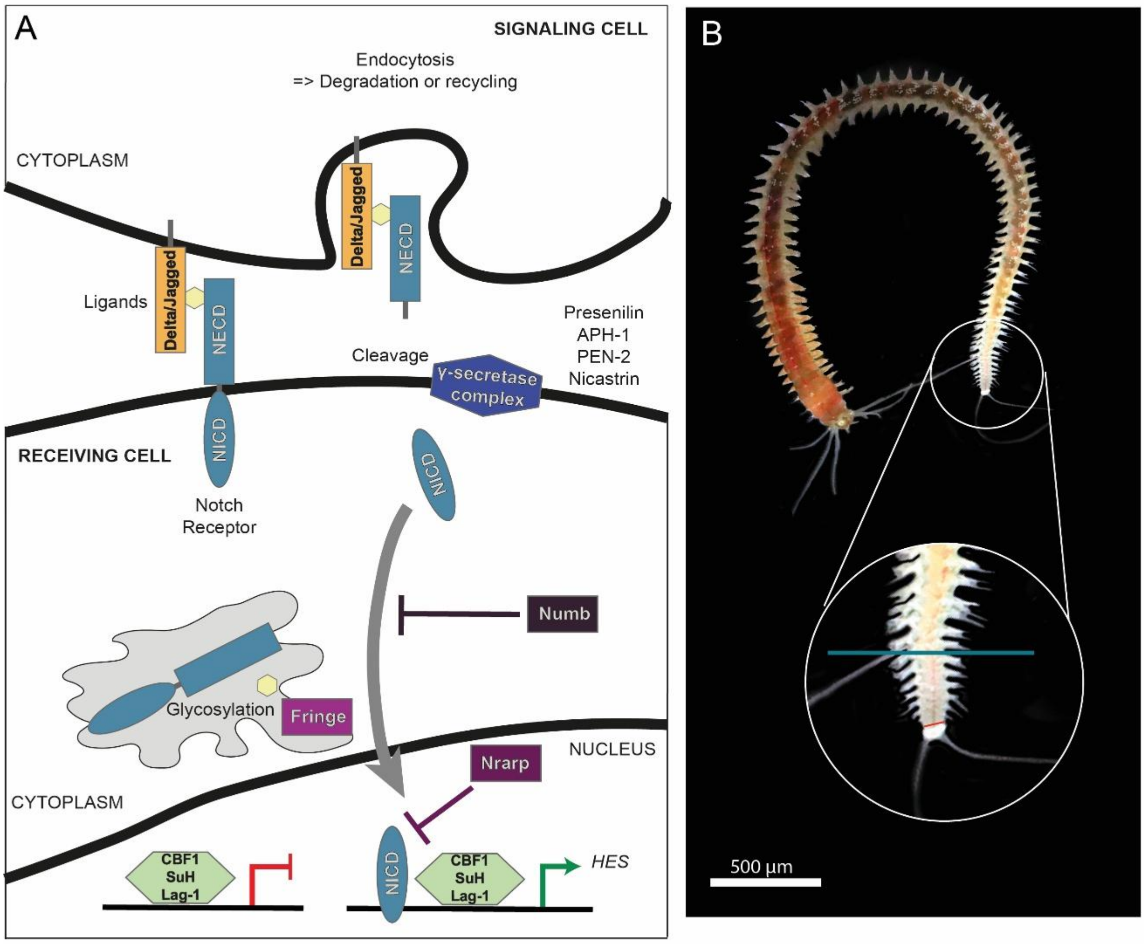

Appendix Figure S1: ***Platynereis dumerilii*** **and the Notch signaling pathway.**

A) Schematic representation of the Notch signaling pathway core components. B) Picture of a *Platynereis* juvenile typically used for regeneration experiments, with a zoom on its posterior part. Amputation plan is in blue.

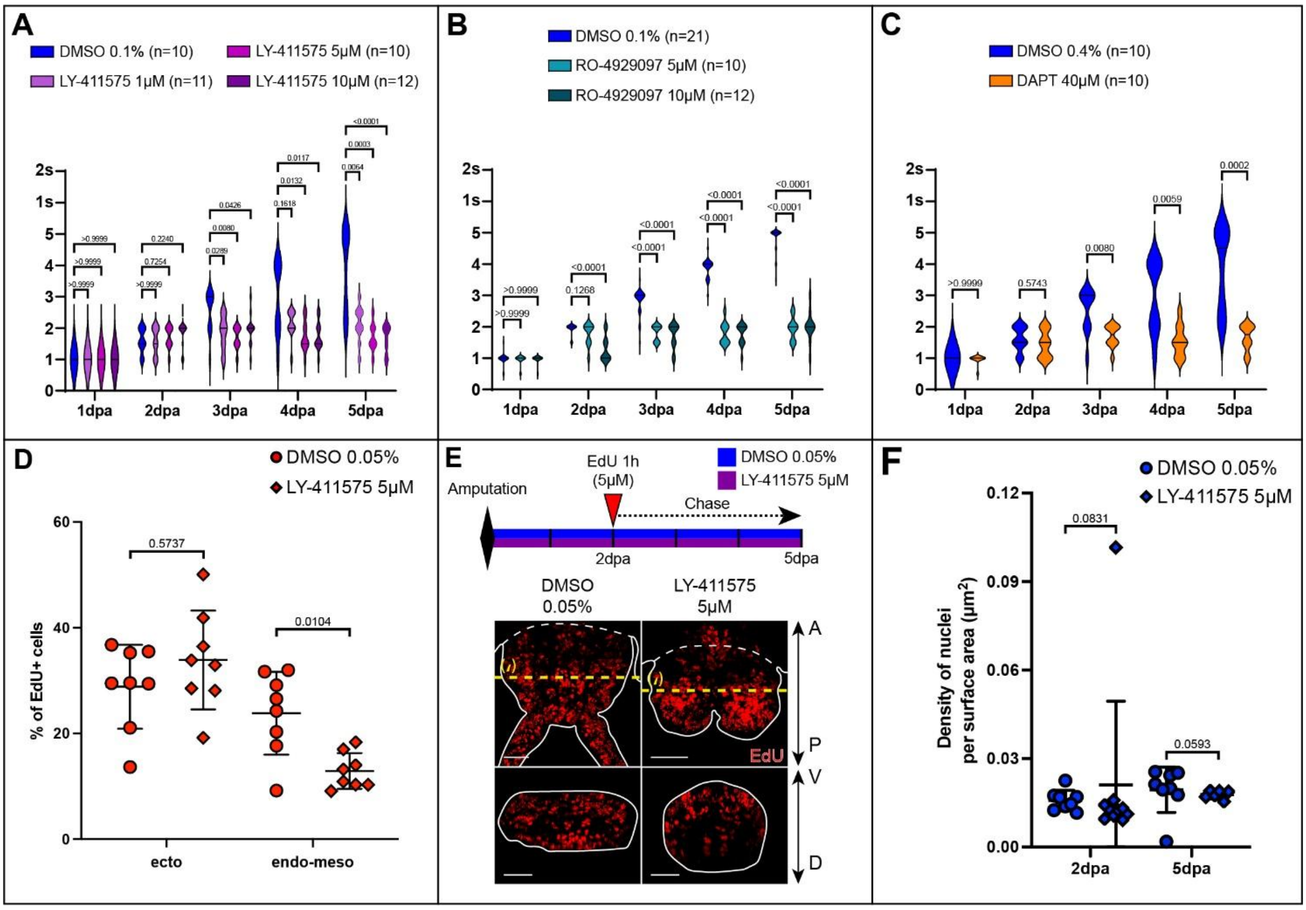

Appendix Figure S2: Determination of the optimal concentrations for Notch pathway inhibitors and additional morphological and cellular effects of LY-411575 treatment during posterior regeneration in the annelid *Platynereis*. (A - C) Violin plots representing the regeneration stages reached by each worm every day for 5 days of treatment. A) LY-411575 treatments at 1, 5 and 10 µM in comparison to control (DMSO 0.1%); B) RO-4929097 treatments at 5 and 10 µM in comparison to control (DMSO 0.1%); C) DAPT treatment at 40 µM in comparison to control (DMSO 0.4%). n = number of worms used per experiment (n ranging from 10 to 12). S = segment. D) Comparison of the proportions of EdU+ cells between the ectoderm ("ecto") and the endo-mesoderm ("endo-meso") at 5 dpa, (n=8 per condition). Data are representative of 2 to 3 independent experiments for A to D. E) Schematic representation of the EdU pulse-and-chase experiment during Notch pathway inhibition and corresponding EdU labelling on LY-411575-treated worms and controls. Ventral views are on top and corresponding virtual transverse sections (along the yellow dotted lines) are at the bottom. Solid white lines delineate the outlines of the samples and white dashed lines correspond to the amputation

planes. Scale bars = 50 µm. Anteroposterior (A/P) and dorsoventral (D/V) axes are represented. F) Comparison of density of nuclei per surface area for LY-411575-treated worms and controls at 2 and 5 dpa (n ≥8).

For data presented in A to D and F, unpaired Mann-Whitney U tests were used for statistical analyses. P-values as well as mean ± s.d. (for D and F) are indicated. dpa = day(s) post-amputation. All images in E come from representative samples of at least four technical replicates. Source data are available online for panel E and in the Dataset EV1 for A, B, C, D and F.

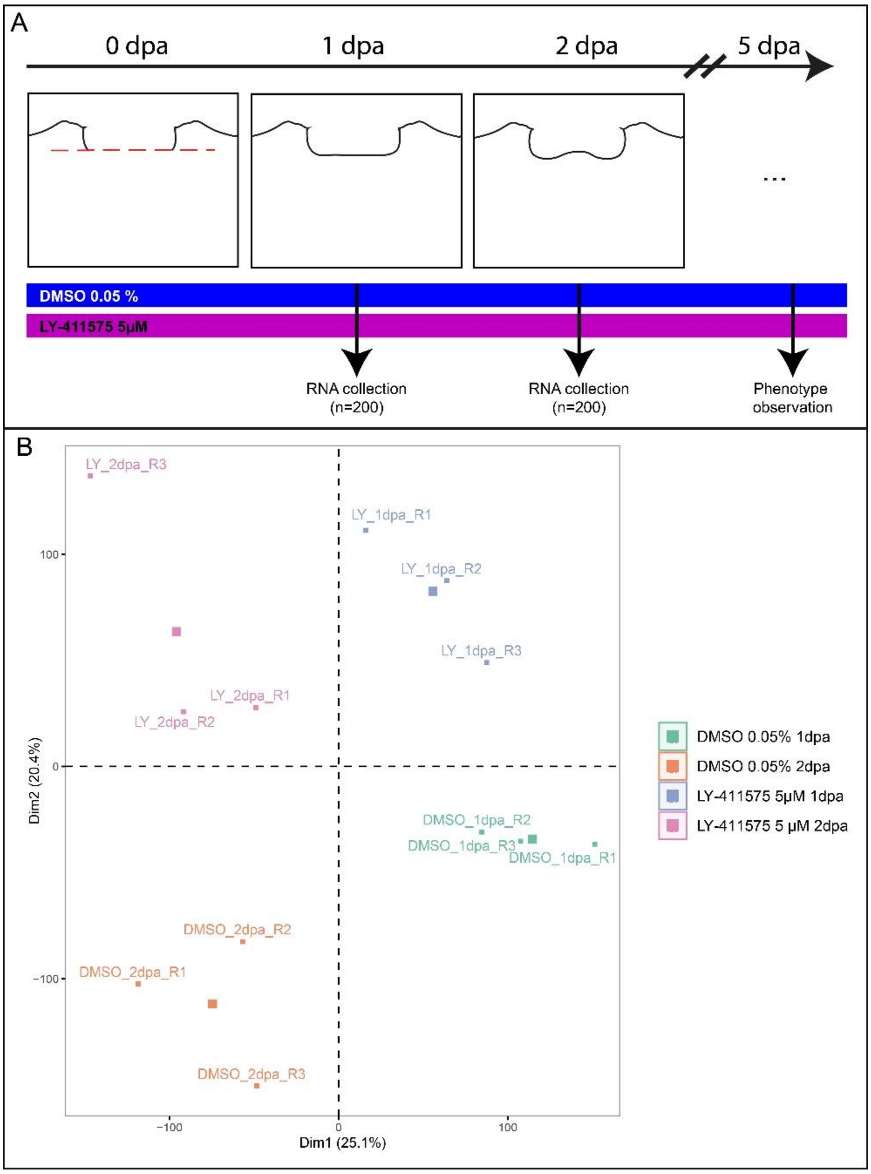

Appendix Figure S3: Schematic representation of the RNA-seq experiment. A) RNA-seq experiment design. Total mRNA was extracted from regenerated parts of LY-411575-treated worms and DMSO controls at 1 and 2 dpa, when the structures are still morphologically comparable. For the 0 and 1 dpa samples, no structure is regenerated (only a wound epithelium is reformed at 1 dpa), so we collected only the hemi-segment abutting the amputation plane. For the 2 dpa samples, we collected both the small bilobate blastema formed at that stage and the hemi-segment abutting the amputation plane. Three replicates (R1 to R3) were produced and about 200 regenerating parts (including a hemi-segment) were collected per replicate. Phenotype was observed at 5 dpa for a couple of animals to ensure the quality of each batch. B) PCA analysis of the 12 RNA-seq samples (4 conditions with 3 replicates). dpa = day(s) post-amputation. Source data are available online.

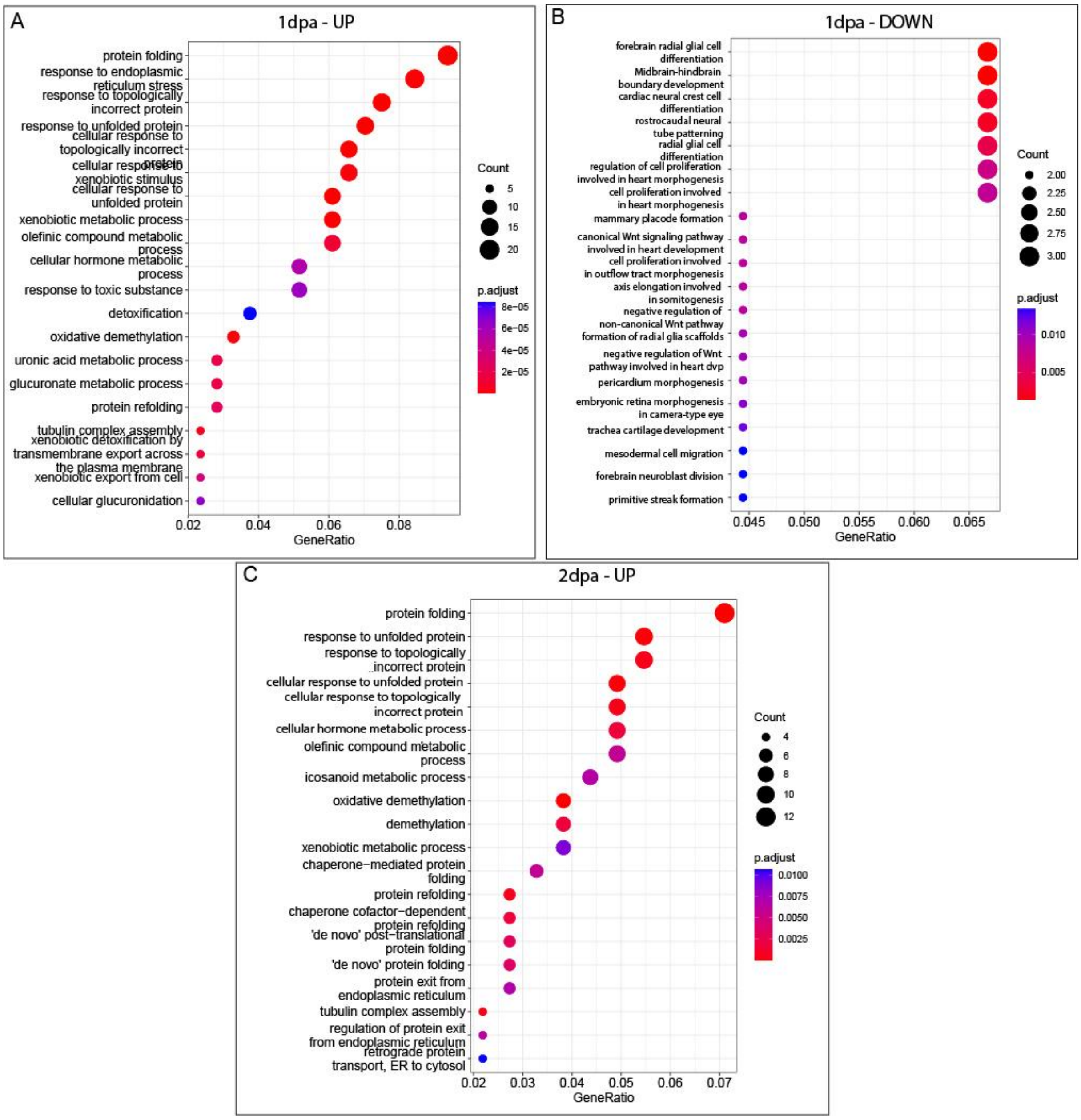

Appendix Figure S4: Gene ontology enrichment analysis of differentially expressed genes (DEGs) between LY-411575-treated animals and controls. Dotplots showing most significant over-represented Biological Process GO terms (Top 20) for DEG upregulated at 1 dpa in LY-treated conditions (A), downregulated at 1 dpa (B) and upregulated at 2 dpa (C). Circles area is proportional to the fraction of transcripts in each condition falling into the corresponding GO term (lines), colors correspond to the adjusted p-value of the enrichment. Gene ratio in x-axis corresponds to the percentage of genes found in each GO term over the total number of genes for each condition. Source data are available online.

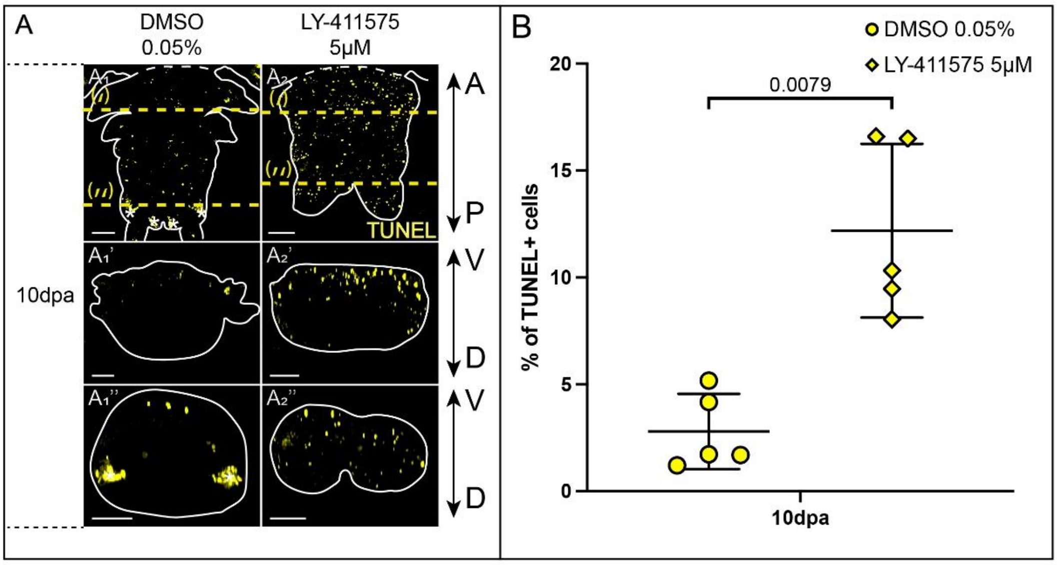

Appendix Figure S5: Notch pathway inhibition during post-regeneration posterior elongation triggers apoptosis. A) TUNEL assay on whole-mount regenerated parts of LY-411575-treated worms from 3 dpa to 10 dpa and DMSO controls at 10 dpa. Ventral views are on the top and corresponding virtual transverse sections along the yellow dotted lines (ʻ and ʻʼ, respectively) are at the bottom. Solid white lines delineate the outlines of the samples and white dashed lines correspond to the amputation planes. White asterisks = non-specific staining from parapodial glands. Scale bars = 50 µm. Anteroposterior (A/P) and dorsoventral (D/V) axes are represented. B) Proportions of TUNEL+ cells between LY-411575-treated worms from 3 dpa to 10 dpa and controls at 10 dpa (n=5). For data presented in B, an unpaired Mann-Whitney U test was used for statistical analyses. P-values are indicated. dpa = day(s) post-amputation. All images come from representative samples of at least four technical replicates. Source data are available online for panel A and in the Dataset EV1 for panel B.

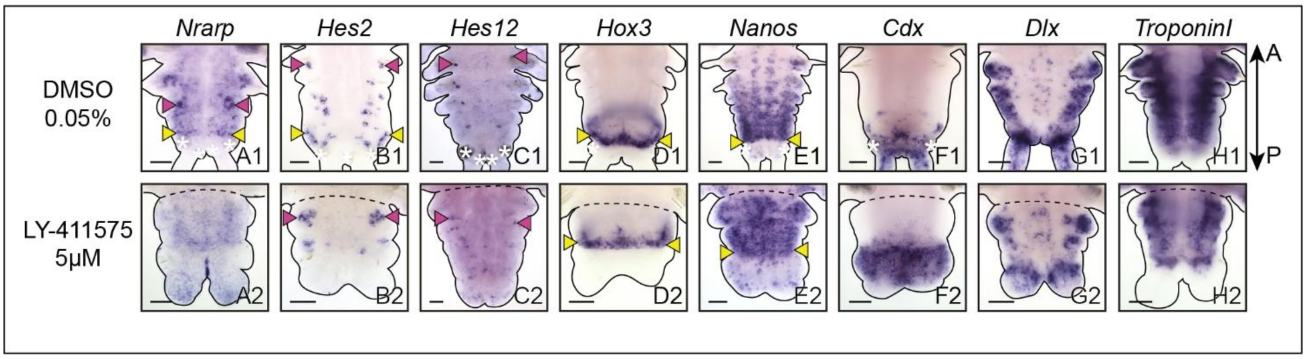

Appendix Figure S6: Effects of Notch signaling pathway inhibition on non-neurogenic tissues during post-regeneration posterior elongation. (A - H) Whole-mount *in situ* hybridizations for *Nrarp* (A) and *Hes* genes (B, C) as well as markers of the growth zone (D), stem/progenitor cells (E), pygidium (F), pygidial cirri and appendages (G) and muscles (H) for LY-411575-treated worms from 3 dpa to 10 dpa and controls at 10 dpa. Ventral views. Solid black lines delineate the outlines of the samples, black dashed lines correspond to the amputation planes. Yellow arrowheads = growth zone involved in posterior elongation of the animals [2]; pink arrowheads = chaetal sacs producing the parapodial bristles; white asterisks = non-specific staining from parapodial glands. dpa = day(s) post-amputation. Scale bars = 50 µm. Anteroposterior (A/P) axis is represented. All images come from representative samples of two biological replicates. Source data are available online.

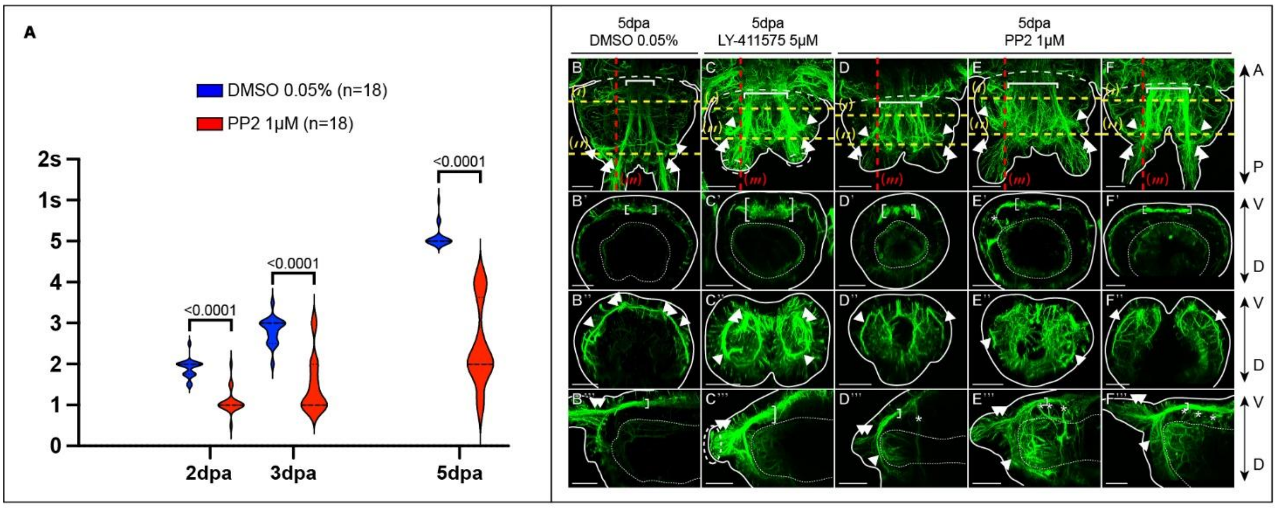

Appendix Figure S7: Inhibiting Src family kinases induces a misshapen axon guidance phenotype that differs from the LY-411575 phenotype. A) Violin plots representing the regeneration stages reached by each worm at 2, 3 and 5 dpa during treatment with the inhibitor of the Src family kinases, PP2 at 1 µM in comparison to control (DMSO 0.05%). Src family kinases are direct downstream effectors of the DCC/netrin pathway. DCC is another substrate of γ-secretase [3] and is involved in axon guidance and neurite outgrowth [4]. n = number of worms used per experiment (n = 18). Data are representative of two independent experiments. (B-F’’’) Acetylated tubulin immunolabelling on whole-mount regenerated parts of DMSO control (B-B’’’), LY-411575 (C-C’’’) and PP2 -treated worms (D-F’’’) at 5 dpa. We observed an important variability for PP2-treated worms and provided images from three samples representative of this diversity. In PP2-treated worms, the ventral nerve chord (VNC, white brackets) is not thickened, there are no distal nerve projections in the pygidium in comparison to LY-411575-treated worms (C, C’’’, dashed ellipse). In contrast, PP2-treated worms present ectopic nerve projections between the VNC and the gut nerve net (D’’’, E’, E’’’, F’’’, white asterisks). Ventral views are on top for each condition and corresponding virtual transverse sections (along (‘) and (’’) in yellow) and sagittal section ((’’’) in red) are at the bottom. In all relevant panels, solid white lines delineate the outlines of the samples, white dashed lines correspond to the amputation planes and white dotted lines delineate the gut. White brackets = ventral nerve chord; white arrowheads = circular pygidial nerve; white double arrowheads = nerves of the pygidial cirri; white dashed ellipse = thick acetylated-tubulin+ *foci*; white asterisks = ectopic nerve projections between the VNC and the gut nerve net. dpa = day(s) post-amputation. Scale bars

= 50 µm. Anteroposterior (A/P) and dorsoventral (D/V) axes are represented. For data presented in A, unpaired Mann-Whitney U tests were used for statistical analyses. P-values are indicated. All images come from representative samples of at least two biological replicates. Source data are available in the Dataset EV1 for panel A and online for panel B to F’’’.
