# Supplementary material for: Multifaceted conserved functions of Notch during post-embryonic neurogenesis in the annelid Platynereis
Source: EMBO Rep. 2026 Apr 1;27(9):2345–68. doi: 10.1038/s44319-026-00731-6 (PMC13172424; doi:10.1038/s44319-026-00731-6)
Supplement: Supplementary file 17 — Expanded View Figures [file 44319_2026_731_MOESM17_ESM.pdf]

Expanded View Figures

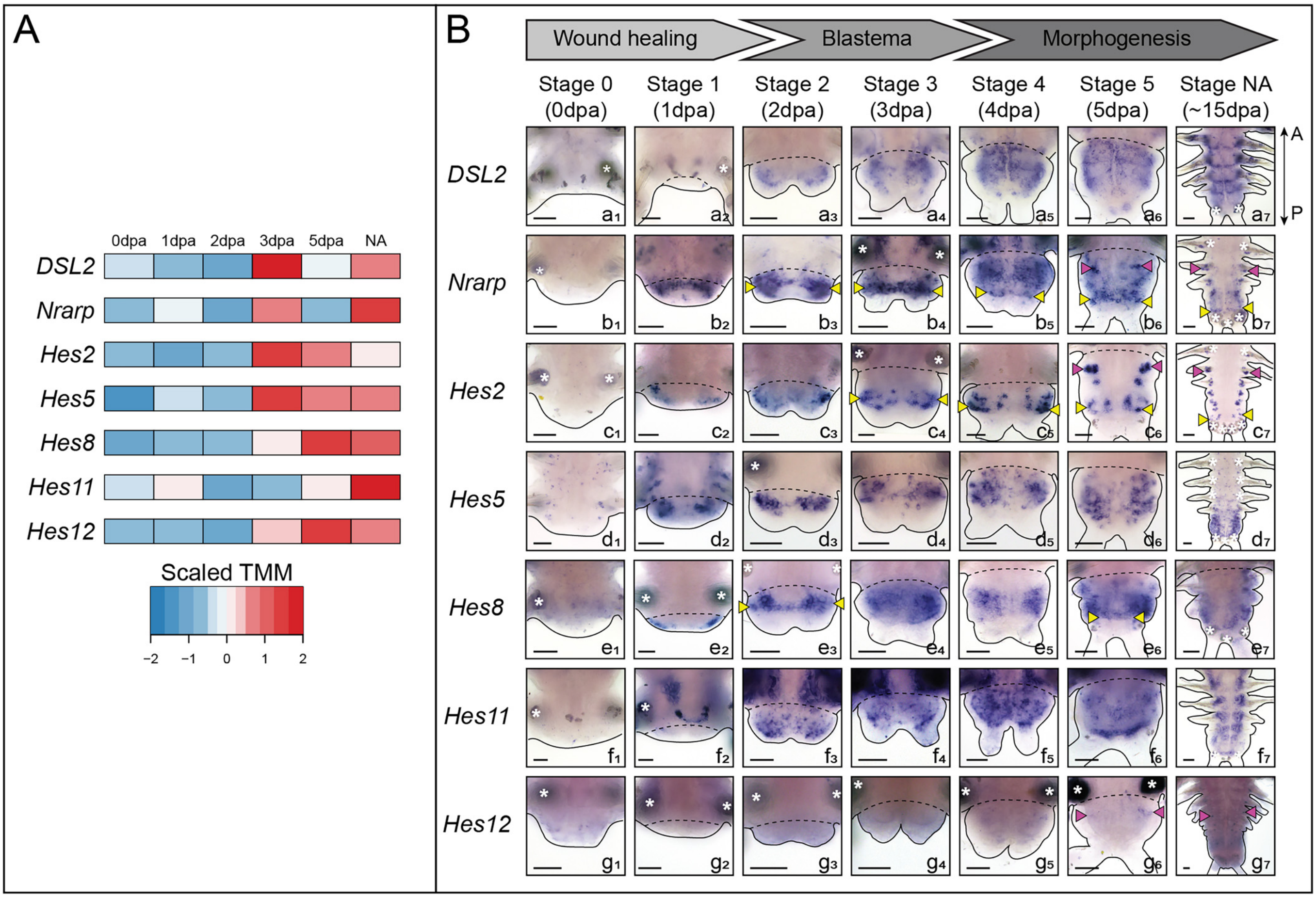

Figure EV1. Dynamic expression of core members of the Notch pathway and its putative target genes in non-neurogenic territories during posterior regeneration.

(**A**) Heatmap representation of expression levels of several Notch components and *Hes* genes during posterior regeneration (Paré et al, 2023). (**B**) Whole-mount in situ hybridizations (ventral views) of Notch components and *Hes* genes expressed in non-neurogenic structures during regeneration. Data information: yellow arrowheads = growth zone involved in posterior elongation of the animals (Gazave et al, 2013); pink arrowheads = chaetal sacs producing the parapodial bristles; white asterisks = non-specific staining from glands. dpa = day(s) post-amputation, NA = non-amputated. Solid black lines delineate the outlines of the samples, black dashed lines correspond to the amputation planes. Scale bars = 50 µm. Anteroposterior (A/P) axis is represented. All images come from representative samples of at least two biological replicates. Source data are available online for this figure.

© The Author(s)

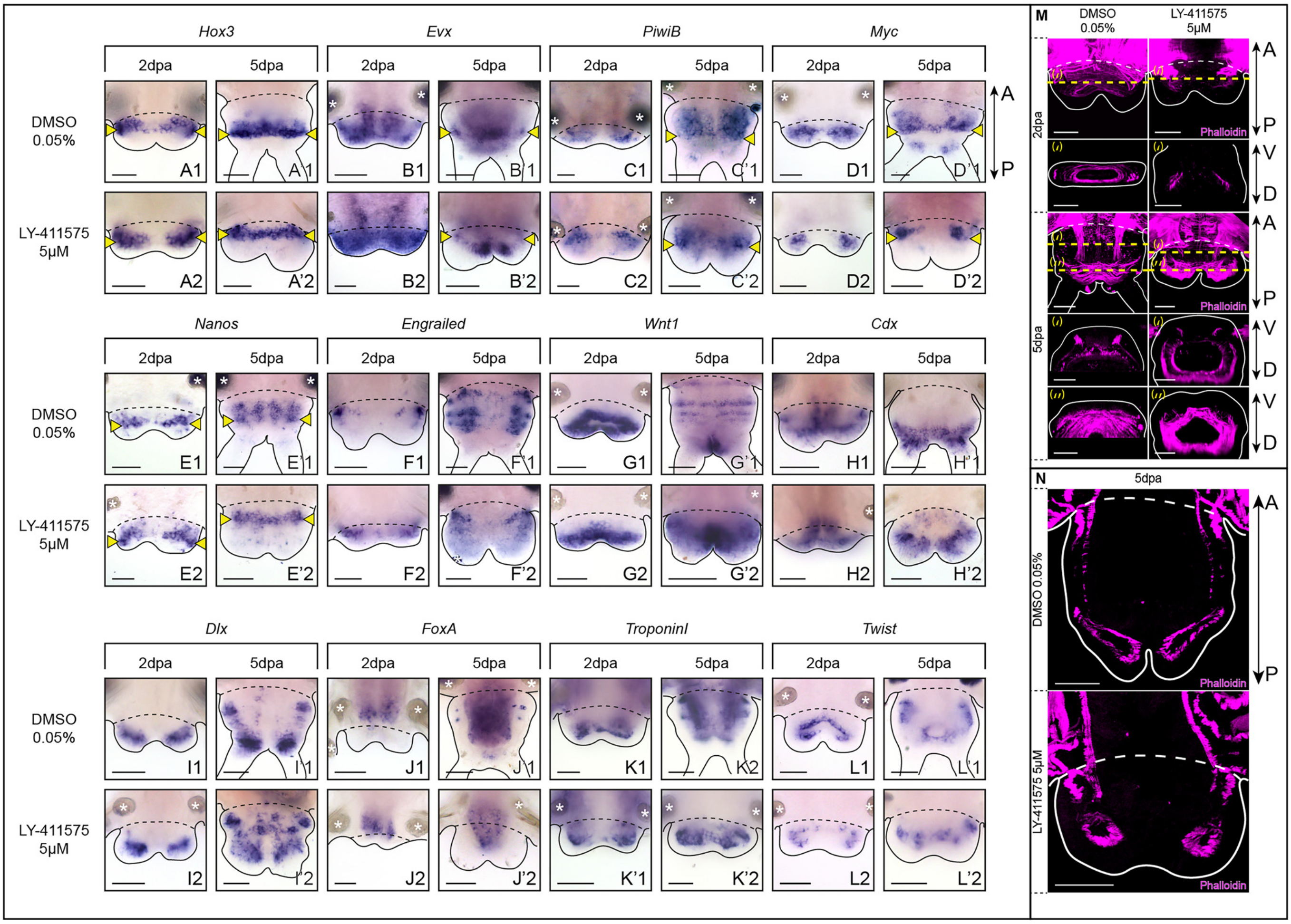

Figure EV2. Effects of Notch signaling pathway inhibition on several tissues during posterior regeneration in *Platynereis*.

(**A–L**) Whole-mount in situ hybridizations for markers of the growth zone (**A**, **B**), stem cells (**C–E**), segmentation (**F**, **G**), pygidium (**H**), pygidial cirri and appendages (**I**), gut (**J**) and muscles (**K**, **L**) for LY-411575 treated worms and controls at 2 and 5 dpa. Ventral views. Solid black lines delineate the outlines of the samples, black dashed lines correspond to the amputation planes. (**M**) Phalloidin labelling on whole-mount regenerated parts of LY-411575-treated worms and DMSO controls at 2 and 5 dpa. Ventral views are on top and corresponding virtual transverse sections (along the yellow dotted lines) are at the bottom. (**N**) Phalloidin labelling on longitudinal cross-sections of LY-411575-treated worms and controls at 5 dpa. (**M**, **N**) Solid white lines delineate the outlines of the samples, white dashed lines correspond to the amputation planes. Yellow arrowheads = growth zone involved in posterior elongation of the animals (Gazave et al, 2013); white asterisks = non-specific staining from parapodial glands. dpa = day(s) post-amputation. Scale bars = 50 µm. Anteroposterior (A/P) and dorsoventral (D/V) axes are represented. All images come from representative samples of at least two biological replicates. Source data are available online for this figure.

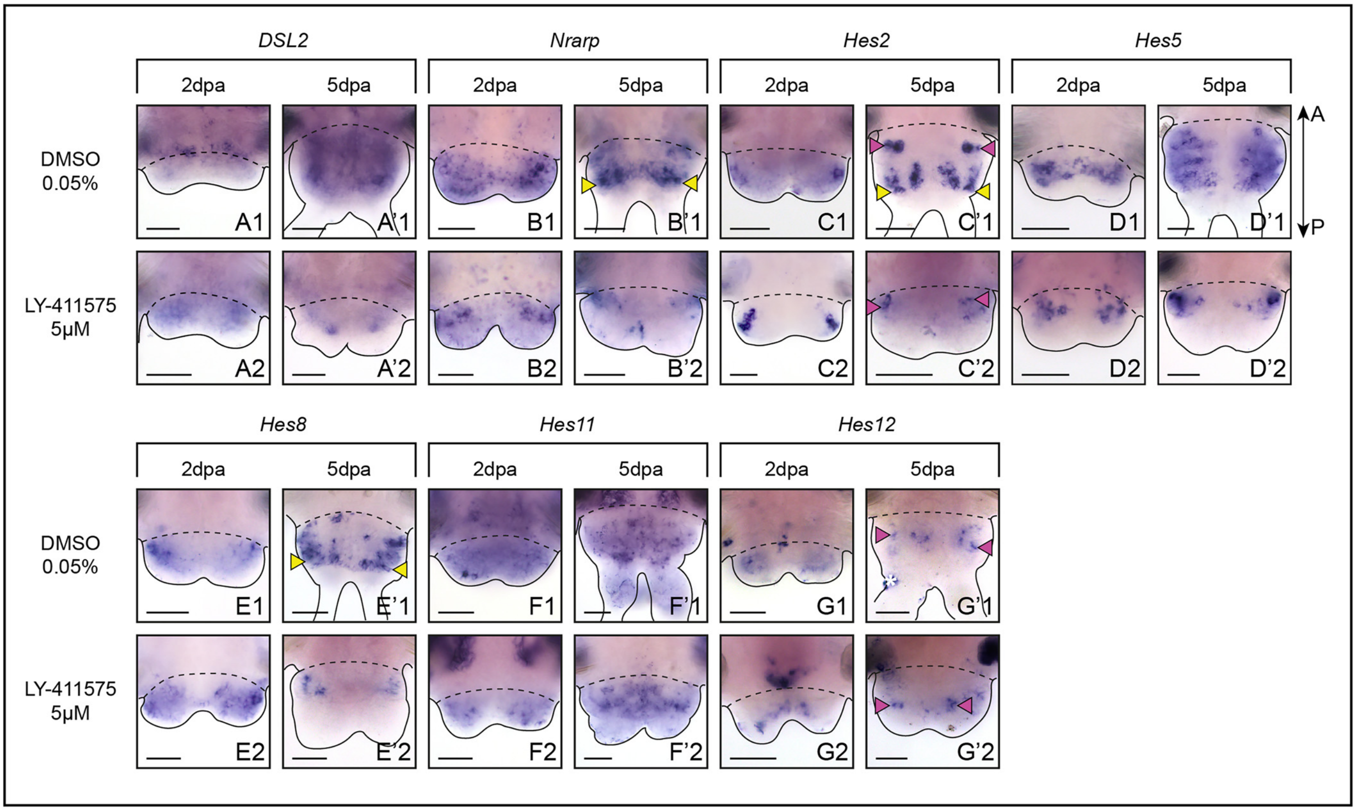

Figure EV3. Impact of Notch pathway inhibition on non-neural territories during posterior regeneration.

(**A–G**) Whole-mount in situ hybridizations of Notch components and *Hes* genes expressed in non-neurogenic structures for LY-411575 treated worms and DMSO controls at 2 and 5 dpa. Ventral views. Solid black lines delineate the outlines of the samples, black dashed lines correspond to the amputation planes. Yellow arrowheads = growth zone involved in posterior elongation of the animals (Gazave et al, 2013); pink arrowheads = chaetal sacs producing the parapodial bristles; white asterisks = non-specific staining from glands. dpa = day(s) post-amputation. Scale bars = 50 μm. Anteroposterior (A/P) axis is represented. All images come from representative samples of at least two biological replicates. Source data are available online for this figure.

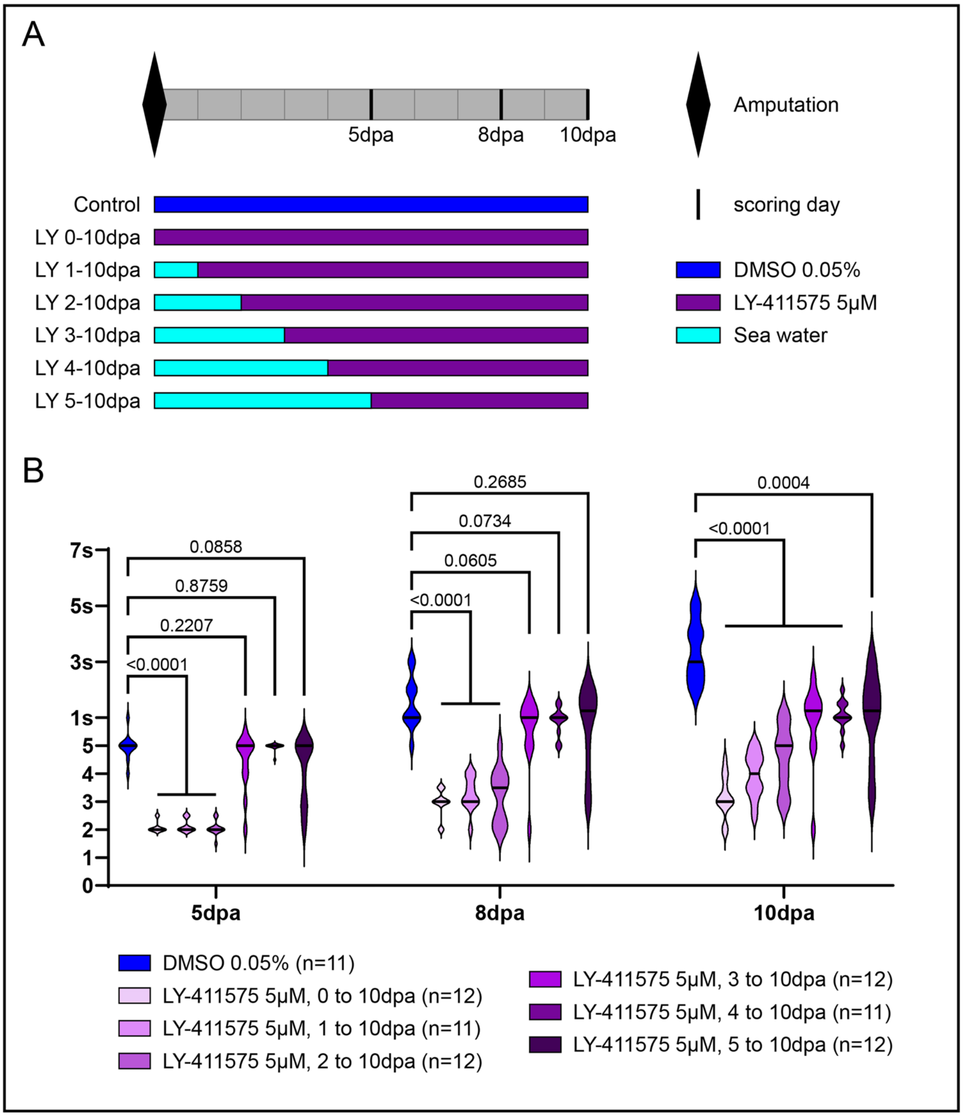

Figure EV4. Morphological effects of different durations of Notch pathway inhibition along posterior regeneration and posterior elongation.

(**A**) Schematic representation of the experiments: six durations of LY-411575 treatment were performed as well as a DMSO control. (**B**) Violin plots representing the stages reached by each worm at 5, 8 and 10 dpa for each treatment. "*n*" represents the number of worms used per condition (*n* ranging from 11 to 12). Data in (**B**) are representative of two independent experiments and unpaired Mann–Whitney *U* tests were used for statistical analyses. *P* values are indicated in the figure and in the Table EV4. dpa = day(s) post-amputation. Source data are available online for this figure.

© The Author(s)

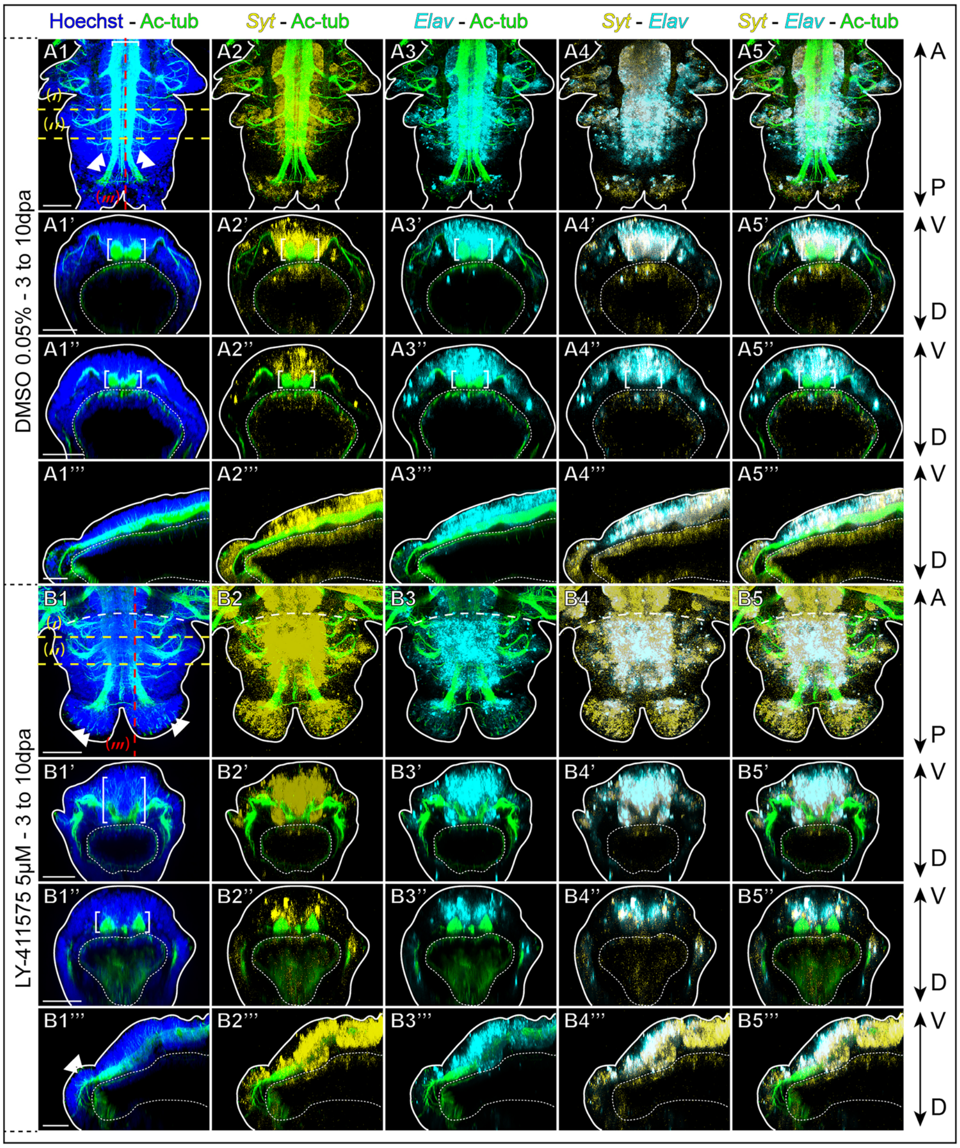

Figure EV5. The neuronal marker *Syt* confirms the phenotype obtained upon Notch pathway inhibition during post-regeneration posterior elongation.

Hybridization chain reactions (HCR) for *Elav* (cyan) and *Syt* (yellow) coupled with immunolabelling for acetylated tubulin (green) and nuclei staining with Hoechst (blue) for controls at 10 dpa (top, **A**) and LY-411575 treated regenerated parts from 3 dpa to 10 dpa (bottom, **B**). Ventral views are on top for each condition and corresponding virtual transverse sections (along (') and ('') in yellow) and sagittal section (along (''') in red) are at the bottom. Solid white lines delineate the outlines of the sample, and white dotted lines delineate the gut. White brackets = ventral nerve cord; white double arrowheads = cirri nerves; blue arrowheads = PNS. dpa = day(s) post-amputation. Scale bars = 50 µm. Anteroposterior (A/P) and dorsoventral (D/V) axes are represented. All images come from representative samples of two biological replicates.
Source data are available online for this figure.

 © The Author(s)
